# A Rare Case of Klippel-Trénaunay Syndrome

**DOI:** 10.7759/cureus.30128

**Published:** 2022-10-10

**Authors:** Akshaya Arasu, Alam Khalil-Khan, Kavin Ilangovan G, Einstein Raju, Lavanya Gunasekaran, Ramprasath Sathiamoorthy

**Affiliations:** 1 Radiology, Chettinad Hospital and Research Institute, Chettinad Academy of Research and Education, Chennai, IND; 2 Academic Unit of Primary Medical Care, The University of Sheffield, Sheffield, GBR; 3 Medicine and Surgery, Government Medical College, Omandurar, Chennai, IND

**Keywords:** bony or soft-tissue hypertrophy, klippel-trénaunay syndrome, painful nevus, venous varicosities, portwine stain

## Abstract

The Klippel-Trénaunay syndrome (KTS) is a rare form of a birth disorder that includes capillary malformation, hypertrophy of bones and soft tissues, and tortuous varicosities, as well as hypertrophy of the capillaries resulting in hemangiomas and port wine discoloration. KTS is also known as angio-osteohypertrophy syndrome and dysplastic angiopathy. In this case report, we describe the case of a 13-year-old female with multiple superficial varicosities on the medial aspect of her left leg since birth. Computed tomography angiogram assessed and identified abnormal venous drainage in the lower limb. Klippel-Trénaunay-Weber syndrome (KTWS) differs from KTS in that KTWS involves arteriovenous malformations.

## Introduction

Maurice Klippel and Paul Trénaunay described Klippel-Trénaunay syndrome (KTS) as a rare variant of congenital birth malformations in 1900 [1], resulting in a triple-featured condition in which small thread-like capillaries (hemangiomas and port-wine discoloration) are malformed, bone and soft-tissue structures are hypertrophied, and the venous system is malformed causing varicosities [2,3]. Hemangiomatosis is the most common cutaneous manifestation of KTS and is usually present from birth. It is estimated that there are two to three cases of KTS per 100,000 live births without sex or ethnic predilection [3]. Neither the etiology nor the pathogenesis of KTS is well understood. A point mutation in the *VG5Q *gene causes KTS, which results in abnormal development of the mesoderm during the intrauterine life of the fetus.

Multiple extremities may be affected by the malformation of the capillary and superficial venous system [4]. Malformations of the capillaries and superficial venous systems may also affect the organs. There have also been reports of deep venous anomalies such as aplasia, aneurysmal dilation, and duplications. Patients are at risk of significant morbidities, including bleeding, deep vein thrombosis, and embolism [5].

## Case presentation

**Figure 1:**
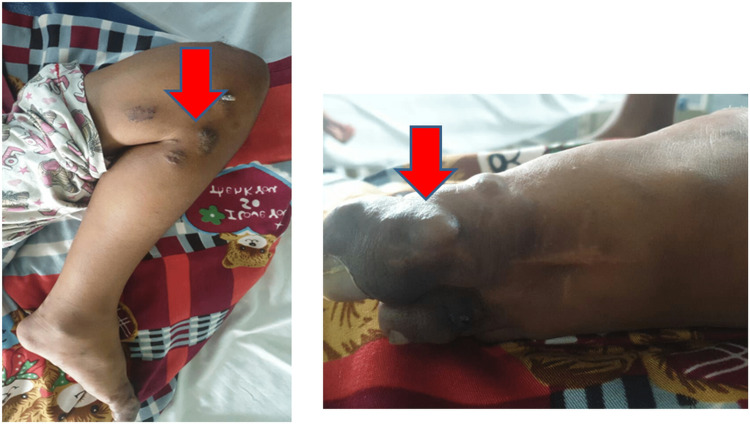
Clinical photograph showing multiple superficial nodules with overlying prominent port-wine stain in the medial aspect of the left thigh and the proximal part of the leg.

A 13-year-old female born of a non-consanguinous marriage presented to the pediatrics department with a history of multiple dilated veins and overlying cutaneous pigmentation in the left lower limb since birth and abdominal pain on the left side (Figure 1).

**Figure 2:**
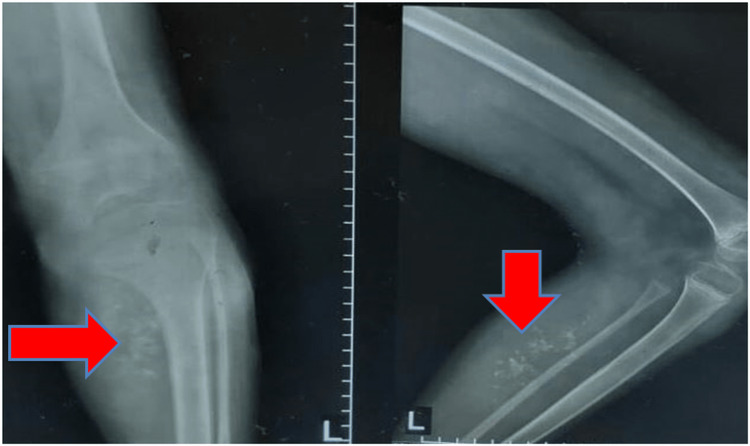
X-ray anteroposterior and lateral view of the left knee joint with femur, tibia, and fibula showing a soft-tissue mass containing phleboliths with underlying bones showing mild osteopenic changes in the medial aspect of the proximal part of the leg.

On physical examination, the malformations were noted in the form of tortuous swelling in the lower limb. The left lower limb had a significant increase in size. While elevating the left limb and in a supine position, the limb returned to its previous size. There was tenderness in the pigmented region. X-ray of the left knee joint with femur, tibia, and fibula showed a soft-tissue mass containing phleboliths along with underlying bones showing mild osteopenic changes in the medial aspect of the proximal part of the leg (Figure 2).

**Figure 3:**
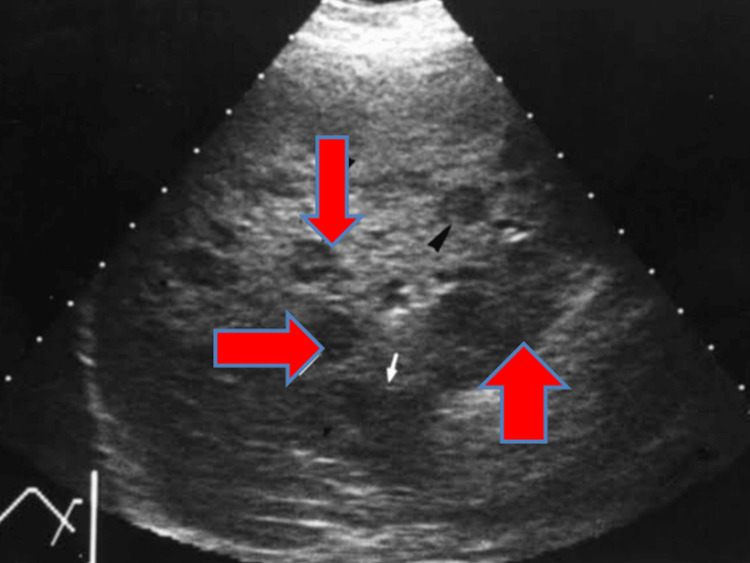
Ultrasound of the abdomen showing an enlarged spleen with clustered cystic spaces without internal vascularity.

Ultrasound of the abdomen showed an enlarged spleen with clustered cystic spaces without internal vascularity measuring 2.4 × 2.3 cm (Figure 3).

**Figure 4:**
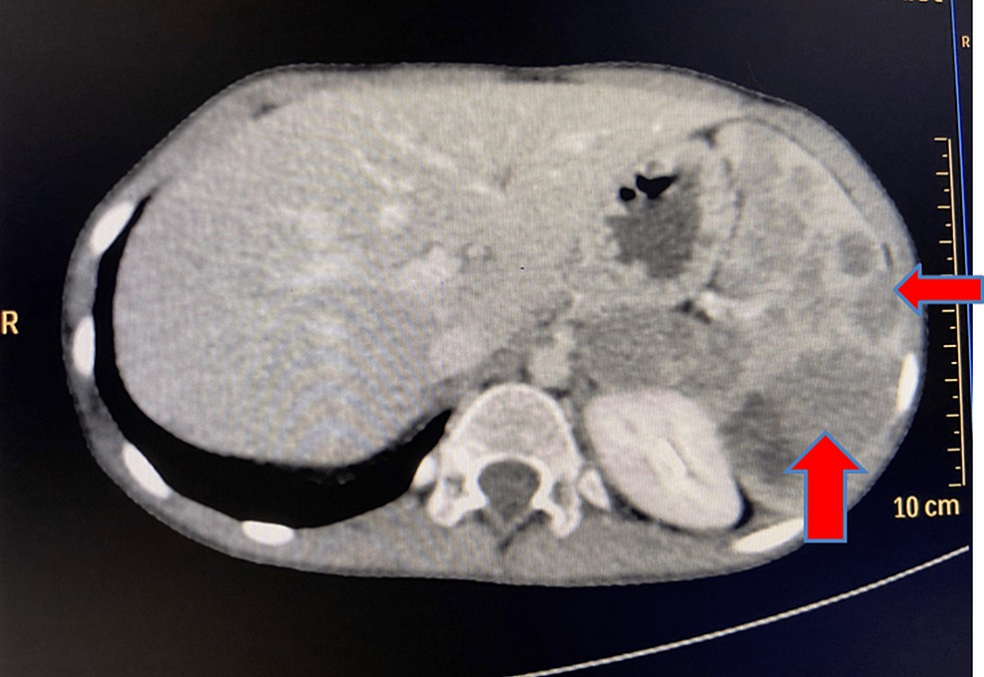
Splenomegaly with multiple geographic hypodense lesions showing progressive enhancement: splenic cystic lymphatic malformation.

Magnetic resonance imaging (MRI) of the lower limb was advised; however, due to the family and the child being averse to undergoing an MRI, a computed tomography (CT) scan of the lower limb with an angiogram was conducted. CT of the abdomen showed an enlarged spleen measuring 13 × 5.9 cm with multiple geographical hypodense lesions seen in the splenic parenchyma along with progressive enhancements due to splenic hemangiomas (Figure 4).

**Figure 5:**
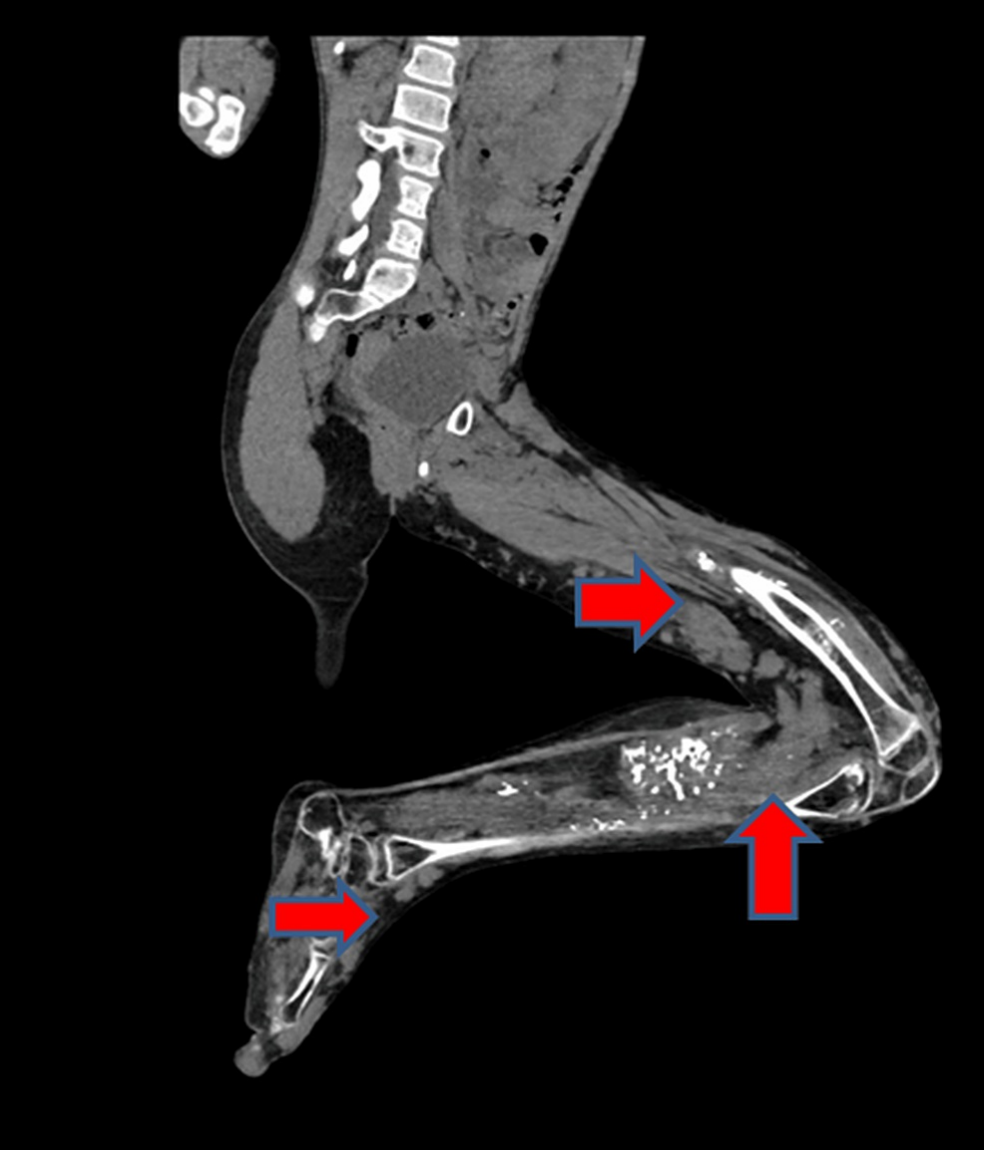
Multiple nodular soft-tissue density lesions are seen predominantly involving the posterior, medial intramuscular compartment of the thigh, calf muscles, and medial subcutaneous aspect of the left lower limb. Several similar lesions are also seen in the foot. Several soft-tissue calcific foci are seen around the knee joint and in calf muscles representing phleboliths.

CT of the lower limb with angiogram showed multiple nodular soft-tissue density lesions predominantly involving the posterior, medial intramuscular compartment of the thigh, calf muscles, and medial subcutaneous aspect of the left lower limb. Several similar lesions were also seen on the left foot. Multiple superficial varicosities were seen in the left leg. Soft-tissue calcific foci were noted around the knee joint and the calf muscles representing phleboliths (Figure 5).

**Figure 6:**
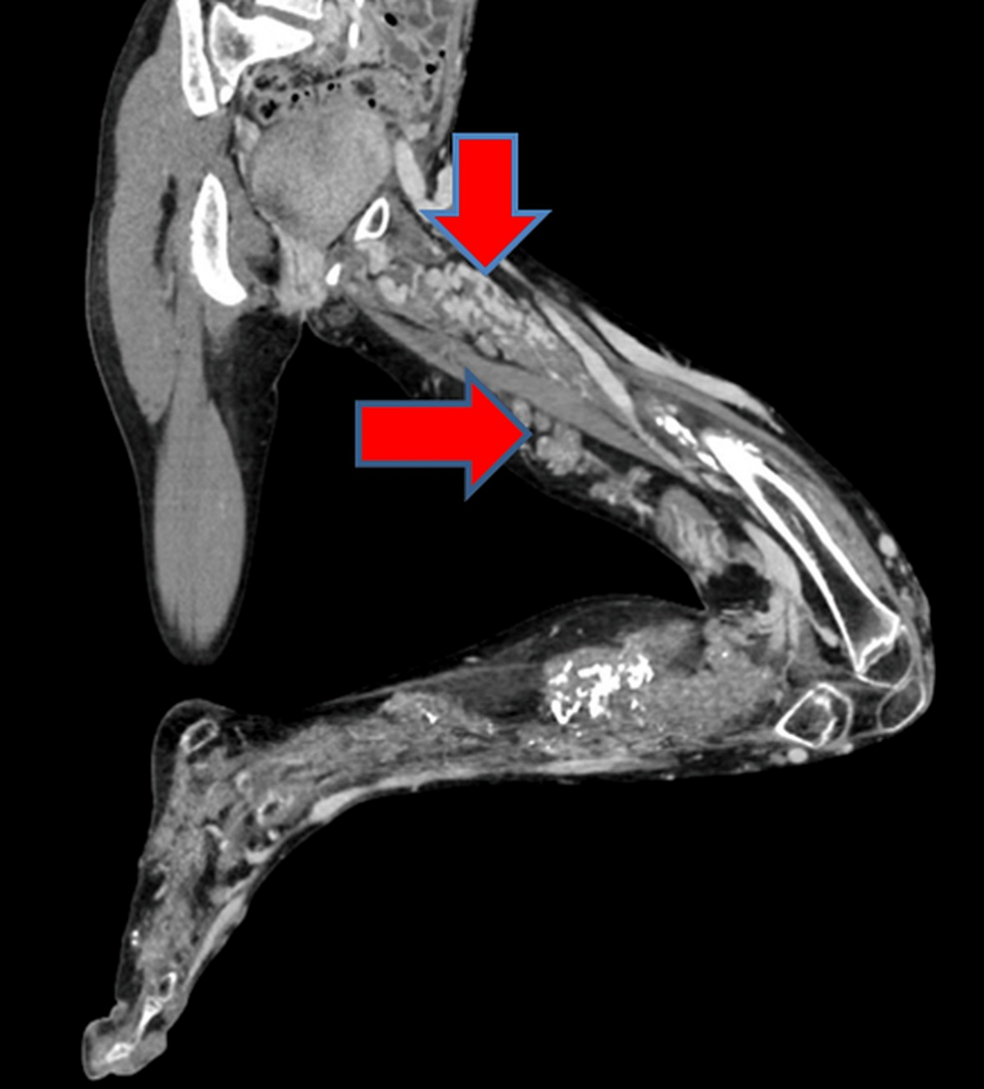
On contrast administration, several of these nodular soft-tissue density lesions are demonstrated to be dilated, tortuous enhancing venous channels.

On the angiogram, several of these lesions were dilated, tortuous enhanced vascular channels. There were no demonstrable arteriovenous channels. The arteries of the left lower limb showed no significant abnormality (Figure 6).

**Figure 7:**
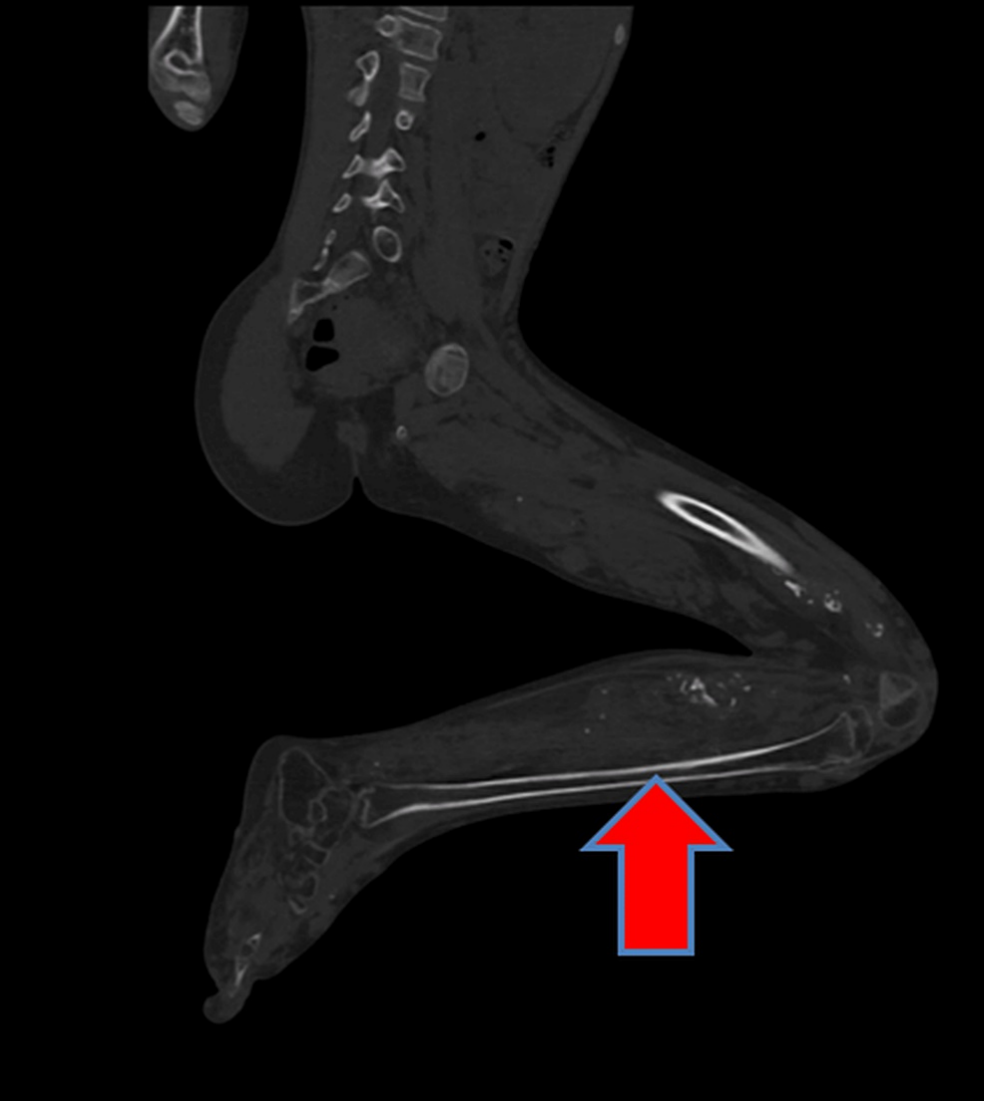
The underlying bones of the left lower limb appear mildly osteopenic. The muscles of the calf appear atrophied with fatty replacement.

The underlying bones of the left lower limb appeared mildly osteopenic. The calf muscles appeared atrophied with fatty replacement. There was no evidence of disproportionate skeletal hypertrophy (Figure 7).

## Discussion

Two French doctors, Maurice Klippel and Paul Trénaunay, demonstrated that three children suffered from cutaneous hemangiomatous lesions along with asymmetrical hypertrophy of soft tissues and bony components in the early 19th century, coining the term “nevus variquece osteohypertrophique” [5]. KTS is a rare syndrome characterized by capillary microvascular malformations and abnormal venous development, resulting in varicosities and hypertrophy of the bone and soft tissue [1,2].

There are several genetic defects that contribute to KTS, including overexpression of the angiogenic factor *VG5Q *and a de novo supernumerary ring chromosome 18 [5,6]. Most cases are sporadic [6]. It has been postulated that there is a primary occlusion of the venous system, resulting in increased intravenous tension, which causes venous varicosities and soft-tissue involvement [5]. Numerous genes overlook the alteration in the balance between angiogenesis and vasculogenesis that results in microvascular abnormality [2,5].

The common features encountered in KTS include discoloration of the dermal aspect of capillaries with varicosities in the Servelle vein which is a marginal vein seen subcutaneously [4]. According to the International Society for the Study of Vascular Anomalies 2018, KTS consists of capillary and venous malformations, limb overgrowth, and lymphatic malformations [4,6,7]. In addition to hyperostosis frontalis interna, rectum and bladder bleeding are other manifestations, which are potentially fatal complications of these malformations and have been reported in less than 5% of cases [8]. The distal colon and rectum are the most frequently reported sites in the gastrointestinal tract. Gut involvement is a severe manifestation of KTS. The initial finding in these cases is painless hematuria [8]. Other imaging findings of KTS include splenic hemangiomas, aneurysms, hemi-megalencephaly, and skeletal manifestations, such as polydactyl/syndactyl, scoliosis, ipsilateral hip dislocations, talipes, meta-tarsus varus [8,9].

Complications related to vascular malformations include dermatitis due to the pooling of venous blood, thrombophlebitis, cellulitis, and worse scenarios, including occlusion of the venous system resulting in consumptive coagulopathies and saddle thrombus in the pulmonary circulation and strain over the heart resulting in its failure [7].

Antenatal ultrasound may diagnose KTS during the late first trimester based on lower limb discrepancies with associated cystic lesions in the subcutaneous plane, fetal hydrops, and polyhydramnios [4,10,11]. An X-ray projection demonstrates elongation of affected limbs due to bone lengthening. Compared to the contralateral leg, this is the main cause of the difference in limb length. KTS is also characterized by the thickening of soft tissues and calcified phleboliths. In contrast to girth discrepancies, length discrepancies are less common. Nevertheless, Parkes-Weber syndrome is associated with differences in limb length [4,7]. T2-weighted MRI sequence shows increased intensity, depicting venous and lymphatic malformation, its deeper extension into the muscular compartments, and hypertrophy of the underlying bones and soft-tissue components [1,3,4,6]. Post-contrast CT and T1-enhanced MRI with contrast of the lower limb shows tortuous varicosities over the superficial aspect of the limbs without any deeper extension is the typical angiographic finding [3,4,6]. A pathognomonic finding is the presence of the Servelle vein in the marginal aspect of the subcutaneous plane over all compartments of the thigh and the outer and lateral aspects of the calf muscles [4,7].

## Conclusions

KTS is characterized by capillary and venous malformations and limb overgrowth, as well as lymphatic malformations. A thorough imaging evaluation is essential to recognize the unusual distribution of varicosities and assess the full extent of the disease. Identifying and treating further complications is part of the further management of the disease.

## References

[1] Martinez-Lopez A, Salvador-Rodriguez L, Montero-Vilchez T, Molina-Leyva A, Tercedor-Sanchez J, Arias-Santiago S (2019). Vascular malformations syndromes: an update. Curr Opin Pediatr.

[2] Abdel Razek AA (2019). Imaging findings of Klippel-Trenaunay syndrome. J Comput Assist Tomogr.

[3] Chagas CA, Pires LA, Babinski MA, Leite TF (2017). Klippel-Trenaunay and Parkes-Weber syndromes: two case reports. J Vasc Bras.

[4] Das R, Kumar I, Verma A, Shukla RC (2019). Spectrum of imaging findings in Klippel-Trénaunay syndrome affecting lower limbs: a report of three cases. Egypt J Radiol Nucl Med.

[5] Harnarayan P, Harnanan D (2022). The Klippel-Trénaunay syndrome in 2022: unravelling its genetic and molecular profile and its link to the limb overgrowth syndromes. Vasc Health Risk Manag.

[6] Jacob AG, Driscoll DJ, Shaughnessy WJ, Stanson AW, Clay RP, Gloviczki P (1998). Klippel-Trénaunay syndrome: spectrum and management. Mayo Clin Proc.

[7] (2020). International Society for the Study of Vascular Anomalies (ISSVA) classification for vascular anomalies. https://www.issva.org/UserFiles/file/ISSVA-Classification-2018.pdf.

[8] Karmacharya RM, Vaidya S, Bhatt S (2022). Klippel-Trenaunay syndrome: case series from a university hospital of Nepal. Ann Med Surg (Lond).

[9] Kharat AT, Bhargava R, Bakshi V, Goyal A (2016). Klippel-Trénaunay syndrome: a case report with radiological review. Med J DY Patil Univ.

[10] Vahidnezhad H, Youssefian L, Uitto J (2016). Klippel-Trenaunay syndrome belongs to the PIK3CA-related overgrowth spectrum (PROS). Exp Dermatol.

[11] Yamaki T, Konoeda H, Fujisawa D (2013). Prevalence of various congenital vascular malformations in patients with Klippel-Trenaunay syndrome. J Vasc Surg Venous Lymphat Disord.

